# Safe and Effective Treatment Choice for Osteoid Osteoma: Computed Tomography-guided Percutaneous Radiofrequency Ablation

**DOI:** 10.7759/cureus.5526

**Published:** 2019-08-29

**Authors:** Cagri Neyisci, Yusuf Erdem

**Affiliations:** 1 Orthopaedics and Traumatology, Gulhane Training and Research Hospital, Ankara, TUR; 2 Orthopaedics, Gulhane Training and Research Hospital, Ankara, TUR

**Keywords:** osteoid osteoma, computed tomography, radiofrequency, ablation

## Abstract

Introduction

Osteoid osteoma (OO) is a painful, benign, bone-forming tumor characterized by a small central nidus surrounded by sclerotic tissue. The aim of the present study was to evaluate the clinical outcome after computed tomography (CT)-guided radiofrequency ablation (RFA) in patients with OO performed from January 2012 to June 2018 and to confirm the safety and efficacy of CT-guided RFA.

Methods

Between January 2012 and June 2018, a total of 63 patients were treated with CT-guided RFA. Pre- and post-treatment pain, further treatment after the RFA procedure, observed complications, and satisfaction with the treatment were recorded for an assessment of clinical effects in all patients. The patients were evaluated with the visual analog scale (VAS) pre-procedure and at three months post-procedure.

Results

Of the patients, 39 were males and 24 were females with a mean age of 21 ± 9.7 (range, 9 to 41) years. The mean follow-up was 16 ± 2.1 (range, 12 to 19) months. The mean duration of the procedure was 34 ± 11.4 (range, 22 to 47) min. All of the patients were diagnosed with OO pathologically. A statistically significant difference was found between the pre-procedural and post-procedural VAS scores (p<0.001). Complications were observed in six patients including one peroneal nerve lesion, three minor skin burns, and two minor skin infections.

Conclusion

This study shows that CT-guided RFA is a safe and effective treatment for OO. It is thought that RFA could be the primary treatment choice for most OO with typical symptoms and radiological findings.

## Introduction

Osteoid osteoma (OO) was first described by Jaffe in 1935 [1]. It accounts for 10%-12% of all benign bone tumors and 3% of all primary bone tumors, is a painful benign bone-forming tumor characterized by a small central nidus surrounded by sclerotic tissue, and has no potential for malignant transformation or metastasis [2-4]. It is usually seen in children and young adults and was also observed most frequently in the second decade. Additionally, it has been rarely observed in patients younger than five years [3-6]. OO is located in the metaphysis and diaphysis of the long bone in more than half of the cases and most commonly occurs in the femur and tibia [4-7].

OO is frequently a nonaggressive lesion and the most common complaint is pain. The pain pattern is characteristic of OO. Generally, the pain pattern begins for months before the diagnosis. The characteristic properties of the pain are: intensive non-mechanical, unrelated to lesion size, being present while relaxing and at night, and rapidly alleviating temporarily by using non-steroidal anti-inflammatory drugs (NSAIDs) or acetylsalicylic acid (ASA). Local swelling, growth disturbance, and bone deformity are other less common symptoms [2,4,8].

OO also has a characteristic radiographic appearance. The radiological diagnosis is based on conventional radiography. The characteristic feature is the nidus, which is a small, oval, central lytic lesion. The nidus is usually less than 15 mm in diameter with a variable quantity of calcification and surrounded by a reactive bone sclerotic rim and adjacent cortical thickening. The nidus can be located most frequently at the periosteal or endosteal surfaces in the cortical or medullary bone. Occasionally, the nidus can be concealed by the very sclerotic reactive bone in the cortex. In such a case, the lesion can be accurately demonstrated in high-resolution computed tomography (CT) imaging [2-4,9-11].

If clinical and imaging features are typical of OO, treatment may be instituted even before histopathological confirmation. The treatment of OO may be performed with intralesional margins because of having limited growth and no malignant potential. The surgical approach is needed only when significant pain impairs normal living. Some lesions have even been reported to resolve spontaneously over time. Due to the potential side effects, long-term treatment with NSAIDs or ASA is not recommended [2,12-13]. There are several treatment options for OO. In the past, the gold standard treatment of choice for OO was the surgical excision of the nidus. This treatment choice could cause a risk of damage to vessels and nerves and an increased risk of fracture due to bone resection. Additionally, intraoperative difficulties with the identification of the lesion and prolonged rehabilitation have been reported. Because of all these, several minimally invasive applications have been developed for the treatment of OO [4,12-17]. Percutaneous radiofrequency ablation (RFA) is one of these applications. Rosenthal et al. initially introduced RFA in 1992 [18]. RFA destroys the nidus with less operative and bone injury, thus eliminating the pain. Local skin area hypoesthesia, breakage of RFA access device, skin burns, and skin infection are the rarely reported complications of RFA [4,13,15,19].

The aim of the present study was to evaluate the clinical outcome after CT-guided RFA in patients with OO performed from January 2012 to June 2018 and to confirm the safety and efficacy of CT-guided RFA.

## Materials and methods

Between January 2012 and June 2018, a total of 63 patients were treated with CT-guided RFA. RFA procedures were performed on all patients with a typical clinical history and a radiologically confirmed diagnosis of OO. All medical records in the patients were reviewed by clinical history, radiological imaging used to confirm the diagnosis, date of procedure, age at the time of treatment, and description of the treatment retrospectively. Pre- and post-treatment pain, further treatment after the RFA, observed complications during the procedure, and satisfaction with the treatment were recorded for an assessment of the clinical effects in all patients. The patients were evaluated with the visual analog scale (VAS) pre-procedure and at three months post-procedure.

Written informed consent was obtained from all individual participants included in the study. The study protocol was approved by Gülhane Military Medical Academy Local Ethics Committee (2015/9). The study was conducted in accordance with the principles of the Declaration of Helsinki.

Surgical procedure

Under general anesthesia, RFA interventions were achieved in a CT room by a radiologist and two orthopedic oncologists. Initially, the nidus and the best skin entry point were located by localizing coronal, sagittal, and axial CT scan using 2 mm collimation. A half-centimeter skin incision was performed on the identified skin entry point. Access to the nidus was gained with a bone biopsy system through the safest and shortest tract under CT guidance. To penetrate the sclerotic cortical bone, a rotatory battery-powered bone biopsy device was inserted percutaneously. A bone biopsy specimen was obtained and used for a histopathological examination. The track was used as an entry for the electrode. During the procedure, the distance between vital structures, such as vessels and nerves, and the electrode was maintained at 1 cm to avoid injuries. The RFA procedure was applied with a 10-12 mm cool-tip RFA system’s electrode for six minutes at 90°C. To provide sufficient application, the electrode can be repositioned according to the obtained necrosis area, the used RFA system, and the lesion size, and an additional procedure may be accomplished. After application, local analgesia was performed at the entry point.

On the postoperative first day, patients were allowed to return to full weight-bearing and their daily activities in accordance with the level of pain. Patients were discharged with oral analgesia at the same procedure day. However, due to the risk of a stress fracture, patients were instructed to avoid arduous activities such as long-distance running for one and a half months.

Statistical analysis

Statistical analysis was performed using IBM SPSS for Mac version 23.0 software (IBM Corporation, Armonk, NY, US) using the paired sample t-test. Descriptive data were expressed as mean ± standard deviation (SD), median (min-max), or number and frequency. A p-value of <0.001 was considered statistically significant.

## Results

**Figure 1:**
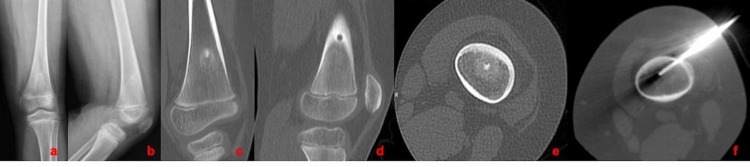
A nine-year-old male patient with OO localized in a) the femur; b) AP and lateral X-ray; c), d) and e) coronal, sagittal, and axial CT view; and f) axial CT view during the RFA procedure OO: osteoid osteoma; AP: anteroposterior; CT: computed tomography; RFA: radiofrequency ablation

**Figure 2:**
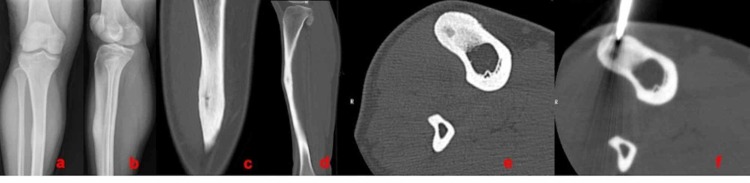
A 20-year-old female patient with OO localized in the a) tibia; b) AP and lateral X-ray; c), d), and e) coronal, sagittal, and axial CT view; and f) axial CT view during the RFA procedure OO: osteoid osteoma; AP: anteroposterior; CT: computed tomography; RFA: radiofrequency ablation

**Figure 3:**
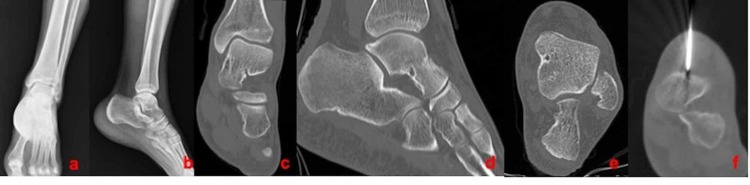
A 24-year-old male patient with OO localized in the a) talus, b) AP and lateral X-ray; c), d), and e) coronal, sagittal, and axial CT view; and f) axial CT view during the RFA procedure OO: osteoid osteoma; AP: anteroposterior; CT: computed tomography; RFA: radiofrequency ablation

Of the patients, 39 were males and 24 were females, with a mean age of 21 ± 9.7 (range: 9 to 41) years. The mean follow-up was 16 ± 2.1 (range: 12 to 19) months. Of the lesions, 31 (49%-18 (58%) metaphysis + 13 (42%) diaphysis) were localized in the femur (Figure 1), 24 (38%- 8 (33%) metaphysis + 16 (67%) diaphysis) in the tibia (Figure 2), two (3%) in the acetabulum, two (3%- 1 metaphysis + 1 diaphysis) in the fibula, two (3%) in the talus (Figure 3), one (2%) in the calcaneus, and one (2%) in the humerus (diaphysis). The mean duration of the procedure was 34 ± 11.4 (range, 22 to 47) min. All of the patients were diagnosed with OO histopathologically. The demographic and clinical characteristics of the patients are shown in Table 1.

**Table 1:** Demographic and clinical characteristics of the patients

Gender	n (%)
Male	39 (62%)
Female	24 (38%)
Total	63 (100%)
Age	Year
Minimum	9
Maximum	41
Mean ± SD	21 ± 9.7
Follow-up	Month
Minimum	12
Maximum	19
Mean ± SD	16 ± 2.1
Location	n (%)
Femur	31 (49%)
Metaphysis	18 (58%)
Diaphysis	13 (42%)
Tibia	24 (38%)
Metaphysis	8 (33%)
Diaphysis	16 (67%)
Acetabulum	2 (3%)
Fibula	2 (3%)
Metaphysis	1 (50%)
Diaphysis	1 (50%)
Talus	2 (3%)
Calcaneus	1 (2%)
Humerus (Diaphysis)	1 (2%)
Total	63 (100%)
Procedure duration	Minute
Minimum	22
Maximum	47
Mean ± SD	34 ± 11.4
Successful RFA procedure	n (%)
First RFA	59 (94%)
Second RFA	4 (6%)
Total	63 (100%)
Pain relief	n (%)
Immediately after the procedure	51 (81%)
Within two days after the procedure	12 (19%)
Total	63 (100%)
Complication	n (%)
Peroneal nerve lesion	1 (2%)
Skin burn	3 (5%)
Skin infection	2 (3%)
Total	6 (10%)

The initial RFA procedure was successful in 59 patients. The procedure was repeated three months later in four patients. Pain relief was observed in 51 patients immediately and in 12 patients within two days after the procedure (Table 1). The mean pre-procedural visual analog scale (VAS) score of the patients was 83.2 ± 6.1 (70-90), and 12.3 ± 11.8 (0-30) three months post-procedural (Table 2). A statistically significant difference was found between the pre-procedural and post-procedural VAS scores (p <0.001). A 91.4% ± 8.3 reduction in VAS scores was detected. According to the pre-procedural and post-procedural VAS scores of the patients, it was found that the pain decreased and patient satisfaction increased significantly with the applied procedure. All of the patients were satisfied with the results of the procedure.

**Table 2:** Comparison of pre-procedural and post-procedural VAS scores VAS: visual analog scale

	Pre-procedural VAS score	Post-procedural VAS score	p-value*
Minimum	70	0	<0.001
Maximum	90	30	
Mean ± SD	83.2 ± 6.1	12.3 ± 11.8	

A major complication, a peroneal nerve lesion was developed in one patient whose lesion was located at the head of the fibula. No additional intervention was performed in the treatment of this patient, only an ankle-foot orthosis (AFO) splint was used and it recovered spontaneously at six months post-procedure. Furthermore, minor complications were observed in five patients. A minor skin burn was developed in three patients and dressing treatment was observed. Two patients experienced minor skin infection at the entry point and treated with oral antibiotics. No additional complications were observed such as iatrogenic fracture, breakage of the RFA access device, or muscle injury (Table 1).

## Discussion

Nowadays, as a result of developing technology, interventional methods, such as RFA with fewer complication rates, which reduces the length of hospital stay rather than surgical treatment, are being developed and used in the treatment of patients with OO [2,4,7,13-15,17-20]. RFA for OO was initially introduced by Rosenthal et al. in 1992 [18]. In this study, we aim to evaluate the clinical outcome after CT-guided RFA in patients with OO and to confirm the safety and efficacy of CT-guided RFA.

OO can be localized in every bone in the body, but it is localized most commonly in the femur [3,5-6]. Similar to the literature, the most common localization was the femur (49%) in our study.

The success rate for the RFA treatment of OO is high and its popularity is increasing worldwide. Recently, CT-guided RFA has been preferred as a safe, effective, and minimally invasive method and as the first treatment method for OO in many clinical centers. The most reliable success predictor is pain relief. The clinical success rate of RFA is in the range of 75%-100% [2,15,21-23]. Rehnitz et al. reported a primary clinical success rate of 96% and after re-RFA, the second clinical success rate of 100% [13]. In the literature, similar studies reported 75%-92% for the primary clinical success rate and 88%-97% for the second clinical success rate [15,22,24]. In the present study, similar to the literature, the primary clinical success rate was found to be 94% and after re-RFA, the secondary clinical success rate was found to be 100%. According to the pre-procedural and post-procedural VAS scores of the patients, a 91.4% ± 8.3 reduction in VAS scores was found in our study. These rates of success compare favorably to those of surgical treatment (88%-97%) [25-28]. Recurrence is associated with younger age, larger lesions, and with the need for multiple needle positions during the procedure. When OO measures exceed 1 cm at its greatest dimension, the use of multiple needle positions during the procedure is often recommended to successfully ablate the lesion. The recurrence rate is less than 15% [2,25-28]. If necessary, we applied multiple needle positions during the procedure. No recurrence was detected within the follow-up period in our study.

The complication rate of RFA in OO is low. Previous studies have reported complications of skin burns, local skin area hypoesthesia, fractures, breakage of the RFA access device, osteomyelitis, vasomotor instability, skin infection, neurovascular injury, tendonitis, and muscular hematoma [4,13,15,19,24-27]. Additionally, chondral damage due to thermal necrosis may develop in the RFA treatment of juxta-articular OOs [29-30]. A local skin burn is the most significant complication. When the active electrode tip is close to the cutaneous surface or in direct contact with the guiding cannulas, the skin burns complication can occur [2,19,29]. In order to avoid the contact of the cannula with the uninsulated active end of the RF ablation electrode, it is important to retract the cannula to a safer distance than 1 cm so that it does not cover the active end of the electrode [2,19,29]. In addition, especially in areas with low subcutaneous soft tissue thickness, such as the anterior tibia, it is recommended to apply prophylactic cold around the cannula inlet region during the procedure [2,19,29]. We paid particular attention that the active electrode tip is not close to the cutaneous surface and/or does not come into direct contact with the guiding cannula during the procedure. However, complications were observed in six patients, including one peroneal nerve lesion, three minor skin burns, and two minor skin infections. No additional complications were observed (Table 1).

In OO, percutaneous ablation techniques other than radiofrequency ablation include alcohol injection and interstitial laser photocoagulation. The disadvantage of alcohol injection is defined as a non-selective effect on tissue and extravasated alcohol affecting the surrounding normal tissue. Laser photocoagulation is quite expensive and simultaneous biopsy cannot be performed in necessary cases [25-26,29-30]. In the event that OO cannot be definitively diagnosed based on clinical findings and imaging, it is preferable to obtain a definite histological confirmation. In patients with expansive or aggressive tumors, a biopsy should always be performed to rule out malignancy. In the present study, all of the patients were diagnosed with OO pathologically.

## Conclusions

In conclusion, the treatment success rate of RFA for OO is high and the rate of procedure failure, complications, and recurrence is low. After treatment, a dramatic improvement in pain, early discharge, a high level of patient satisfaction, and return to daily life without pain is expected. In the treatment of osteoid osteoma at appropriate locations, RFA has got ahead of surgical treatment. However, skin burns that may develop during the procedure constitute a serious problem, and it is recommended to pay particular attention to avoid this complication. This study shows that CT-guided RFA is a safe and effective treatment for OO. It is thought that RFA could be the primary treatment of choice for most OO with typical symptoms and radiological findings.
